# Work-related risk factors for Carpal Tunnel Syndrome among Majmaah University female touchscreen users

**DOI:** 10.12669/pjms.35.6.1529

**Published:** 2019

Dear Editor,

I have read the original article entitled “Work-related risk factors for Carpal Tunnel Syndrome among Majmaah University female touchscreen users” by Walaa Sayed Mohammad,^1^ published in Pakistan Journal of Medical Sciences 2019;35(5). I want to congratulate the authors for this successful clinical study and contributing to the evidence-based practice in rehabilitation.

There are certain methodological concerns in the article before we use the results of the study in clinical practice or to identifying the risk group. Firstly, the electrophysiological properties of the medial nerve change throughout the course of the day. Therefore, symptoms perceived in the morning will not correspond to the symptoms perceived by the same subject in the evening, not considering the Diurnal variation of electrophysiological symptoms associated with carpal tunnel syndrome^2^ would reduce the internal validity of the study. Secondly, academicians, teaching support staff and students undergo a stressful phase before and during the semester examination.^3^ These stressful activities include typing an assignment on the computer, evaluating a written answer from students. Therefore a specific period needs to be identified for subjects either stressful or non-stressful for a given population in the sample.

Following are the recommendations for future research.

1. The authors must assess the entire subject at a specific time to avoid diurnal variation and to increase the test-retest reliability of assessment tools used in the study.
2. The psychological state of the individual is likely to influence the excitability of nerves and flexibility of the musculoskeletal system. Therefore a prior anxiety and depression assessment of subjects are mandatory.

***Response from the authors:***

1. This point was considered during the assessment of all participants. Therefore, all participants were assessed at the same specific time (particularly, during the morning hours 9-11 am). Consequently, the test-retest reliability of assessment was high.
2. All participants were assessed throughout the semester period (away from the final exam period) where the stresses were assumed to be unified and fairly equal.

Dr. Walaa Sayed Mohammad Associate Professor of Biomechanics Physical Therapy Department

*College of Applied Medical Sciences Majma’ah University Majmaah, Saudi Arabia*.

**Efficacy of Paraffin Wax Bath with and without joint mobilization techniques in rehabilitation of post-traumatic stiff hand**

This refers to the above mentioned study by Fozia Sibtain and colleagues published in Pakistan Journal of Medical Sciences in 2013 Vol.29 No 2. 647-650. The standard deviation values given in Table-II are not understood properly. I do not understand why the standard deviation is expressed in the format “x.xxx + x.xxx”.I would be very grateful if you could confirm this.

Jae Hyun Jung Division of Rheumatology, Department of Internal Medicine, Korea University Ansan Hospital, Korea. E-mail: mcfriend82@naver.com

***Response from authors:*** It is actually mean and stranded deviation given in the Table-II of the study outcomes at the completion of intervention program of six weeks duration in both the groups. It is suggested to correct the heading and change it from std, deviation to mean (std. deviation) and add ± which is currently + only. The remaining table is fine.

***Prof. Dr. Syed Shakil Ur Rehman*.**

***syedshakilurrehman@yahoo.com***
